# A Review on the Ethnomedicinal Usage, Phytochemistry, and Pharmacological Properties of Gentianeae (Gentianaceae) in Tibetan Medicine

**DOI:** 10.3390/plants10112383

**Published:** 2021-11-05

**Authors:** Xiaofeng Chi, Faqi Zhang, Qingbo Gao, Rui Xing, Shilong Chen

**Affiliations:** 1 Northwest Institute of Plateau Biology, Chinese Academy of Sciences, Xining 810008, China; xfchi@nwipb.cas.cn (X.C.); fqzhang@nwipb.cas.cn (F.Z.); qbgao@nwipb.cas.cn (Q.G.); xingrui@nwipb.cas.cn (R.X.); 2 Qinghai Provincial Key Laboratory of Crop Molecular Breeding, Xining 810008, China

**Keywords:** Gentianeae, ethnomedicinal usage, phytochemistry, pharmacological properties, Tibetan medicine

## Abstract

Gentianaceae is a large plant family and is distributed worldwide. As the largest tribe in Gentianaceae, Gentianeae contains 939–968 species, and the Qinghai-Tibet Plateau and adjacent areas are the main centers of diversity for Gentianeae. Species from the Gentianeae are widely used in traditional Tibetan medicine. In this review, a systematic and constructive overview of the information on botany, ethnomedicinal usage, phytochemistry, and pharmacological properties of Gentianeae in Tibetan medicine is provided. The results of this study are based on a literature search, including electronic databases, books, websites, papers, and conference proceedings. Botanical studies showed that Gentianeae includes the subtribe Gentianeae and Swertiinae, and several new genera and taxa have been identified. Approximately 83 species from Gentianeae were used in Tibetan medicine, among which *Gentiana* and *Swertia* constituted the largest number of species with 42 and 24 species, respectively. The species from Gentianeae are mainly used as Bangjian (སྤང་རྒྱན།), Jieji (ཀྱི་ལྕེ།), Dida (ཏིག་ཏ།), and Ganggaqiong (གང་གྰཆུང་།) in Tibetan medicine with different clinical applications. More than 240 formulas were found containing Gentianeae species with different attending functions. Phytochemical studies showed that the main active components of Gentianeae species are iridoids, xanthones, flavonoids, and triterpenoids. The bioactivities of plants from Gentianeae include hepatic protection, upper respiratory tract protection, joint and bone protection, glucose regulation, antibacterial, antioxidant, anticancer, and antiviral effects. This review will provide a reference for future research on natural resource protection, plant-based drug development, and further clinical investigation.

## 1. Introduction

Tibetan medicine has a long history of being rich in active components, which embodies the precious experience of Tibetans in their long-term struggle against diseases. Moreover, Tibetan medicine is based on a unique theoretical system and strong national characteristics. The origin of Tibetan Medicine is extremely complicated. Tibetan medicine is not only a syncretism of Chinese, India, and Persia medicinal opinions but also has unique historical origins, which may stem from pre-Buddhist sources or may have Ayurvedic origins [1,2,3]. In 2006, Tibetan medicine was included in the first batch of the national intangible cultural heritage list of China.

The history of Tibetan medicine dates back to 200 BC (the reign of Nyatri Tsenpo [གཉའ་ཁྲི་བཙན་པོ།], the first governor of Tibet). The famous assertion of Tibetan medicine, “Poisonous, there is medicine” was proposed, which foreshadowed the budding of Tibetan medicine [4]. In 641 BC, the governor of Tibet, Songtsen Gampo (སྲོང་བཙན་སྒམ་པོ), married Princess Wen Cheng, princess of the Tang dynasty. Several medical books, diagnostics, herbal prescriptions, and medical instruments were brought to Tibet, which significantly promoted the development of Tibetan medicine [5]. Around the middle of the 8th century, a classic book on Tibetan medicine, “Sman-dpyad Zla-bavirgyal-po” (སྨན་དཔྱད་ཟླ་བའི་རྒྱལ་པོ།) was published, in which more than 440 types of botanical medicine, 260 types of animal-based medicine, and 80 types of medicine of mineral origin were recorded, and 30 materia medicas were unique to the Qinghai-Tibet Plateau [6]. At the end of the 8th century, Yuthok Yontan Gonpo (གཡུ་ཐོག་རྙིང་མ་ཡོན་ཏན་མགོན་པོ་) wrote “Dpl Ldn rGyud Bzhi” (༅༅།།བདུད་རྩི་སྙིང་པོཡན་ལག་བརྒྱད་པ་གསང་བམན་ངག་གི་རྒྱུད་ཅེས་བྱ་བ་བཞུགས་སོ།།), in which 1002 types of medicines were included and divided into 8 categories [7]. In 1835, the book “Shel Gong Shel Phreng” (ཤེལ་གོང་ཤེལ་ཕྲེང་།) was written by Devu Dmr Dge Bshes Bstn Vdzin Phun Dzogs (དེའུ་དམར་བསྟན་འཛིན་ཕུན་ཚོགས།), which is regarded as the greatest achievement in Tibetan medicine [8]. It lists 2294 drugs (1006 botanical drugs, 448 drugs from animal origin, and 840 drugs of mineral origin) classified into 13 categories. Most importantly, the shape, color, use, and habitat of each drug are described and many previous studies from the literature have been cited.

More than 2400 types of Tibetan medicine were included in ancient books and records and include more than 2170 botanical drugs, 210 drugs of animal origin, and 50 mineral drugs [9]. Among botanical medicines, more than half were distributed in the Qinghai-Tibet Plateau, especially a variety of alpine plants were widely used, such as “Snow Lotus”, “Himalayan poppy”, “Rhododendrons”, and “Gentians” [10,11,12]. Unique growing environments such as high altitude, long exposure to strong ultraviolet rays, cold, drought, and lack of oxygen partially contributed to the distinctive therapeutic effects of herbals. However, the cultivation of medicinal herbs was difficult in this area owing to the unique geographical conditions and climatic characteristics, and most herbal medicines were collected from the wild by excessive mining [13].

In terms of the varieties of medicines used, there is currently no professional market for Tibetan medicinal materials as the production of these materials mainly depends on the collection from wild resources. Most medicinal materials used in Tibetan medical institutions and pharmaceutical enterprises in various locations are self-equipped to collect the local, wild medicinal resources. Approximately 80% of medicinal materials consist of varieties unique to Tibetan medicine and are produced in Tibet, Qinghai, Gansu, Sichuan, and Yunnan. Different regions in Tibet use different medicinal materials, the source is closely related to the condition of local species of medicinal resources, and the significant characteristics of the “regional”, “homonym”, “synonyms”, “local learning supplies”, or substitute more phenomenon are extremely common [14]. Owing to the particularity of the traditional Tibetan cultural background and the relatively weak scientific and technological strength of Tibetan medicine, research on variety arrangement, quality standards, resource protection, and the utilization of Tibetan medicinal materials lag considerably and the quality standard of the products used in this system of medicine is far from perfect [15]. Thus, strengthening the sorting of Tibetan medicine species and establishing a standard system are important aspects to regulate their use in a clinical setting, guide pharmaceutical enterprises to synthesize qualified, safe, and effective drugs, improve the ability of drug supervision, protect, and ensure the rational use of Tibetan medicinal resources, and promote the cultural exchange of Tibetan medicine.

Gentianaceae is a large plant family with a global distribution [16]. It is primarily distributed in the temperate regions of the Northern hemisphere. There are about 103 genera and approximately 1600 species worldwide. Gentianaceae contains six tribes in which Gentianeae takes up more than half of the species in Gentianaceae. A total of 15 genera and 410 species of Gentianeae are found in China, of which 2 genera and 251 species are endemic [17]. The Qinghai-Tibet Plateau and adjacent areas are the main centers of diversity for Gentianeae [18]. Therefore, species from the Gentianeae tribe are one of the most widely used medicinal plants in traditional Tibetan medicine.

The potential of Tibetan medicine in healthcare is being recognized; thus, Gentianeae is attracting increased attention from medical professionals. A systematic review of the traditional use of this tribe and elucidating the chemical composition and pharmacological effects of Gentianeae will provide a powerful reference for the in-depth study, rational use, and effective protection of this valuable resource.

## 2. Data Collections

All data in this review were summarized from references, including scientific journals, book chapters, or dissertations. All the references were searched in several electronic databases, including PubMed, Web of Science, Scopus, ScienceDirect, CNKI (https://oversea.cnki.net/index/), Google Scholar (https://scholar.google.com/), and Baidu Scholar (https://xueshu.baidu.com/) with “Gentianeae (Gentianaceae) in Tibetan Medicine” as keywords without any other restrictions. Subsequently, literature closely related to botanical study chemical composition, traditional uses, and pharmacological properties were screened. In addition, the classification and geographical distribution of Gentianeae plants were searched in Plant Plus of China (https://www.plantplus.cn/cn), Global Biodiversity Information Facility (GBIF, https://www.gbif.org/) and The Plant List (http://theplantlist.org/). The last accessed date to the links mentioned above in order to acquire data was 31 May 2021.

## 3. Results

### 3.1. Botanical Studies of Gentianeae

Gentianeae has the largest number of species amounting to 939–968, which account for about 57% of Gentianaceae [16]. Gentianeae includes the subtribe Gentianeae and Swertiinae. The morphology, palynology, flower anatomy, and chromosome characteristics of Gentianeae were comprehensively summarized, and the classification history was analyzed [16,19,20]. On the basis of classical morphological classification, phylogenetic studies of Gentianeae have made a lot of progress with the development of molecular systematics, and the phylogenetic relationships among groups have become increasingly clear. Several new genera and taxa have been identified.

#### 3.1.1. Subtribe Gentianeae

Struwe et al. divided the subtribe Gentianeae into three genera, *Gentiana*, *Crawfurdia*, and *Tripterospermum* based on the analysis of *trn*L-intron, *mat*K, and ITS sequences combined with morphological studies [16]. Ho et al. published a new genus, *Metagentiana*, based on morphological characteristics [21]. Faver et al. separated the genus *Sinogentiana* from *Metagentiana* and the genus *Kuepferi* from *Gentiana* based on morphological characteristics and molecular phylogeny [22]. Thus, the current subtribe Gentianeae consists of six genera, namely, *Gentiana*, *Metagentiana*, *Kuepferia*, *Crawfurdia*, *Sinogentiana*, and *Tripterospermum*. *Gentiana* (350 species) is the largest genus of Gentianaceae that originated in the Qinghai-Tibet Plateau and differentiated with the rise of the plateau and with climatic changes [18]. *Kuepferia* (14 species) and *Sinogentiana* (2 species) prefer cool and dry habitats and are rather conserved niches. Despite a tendency for niche evolution, *Crawfurdia* (18 species) and *Metagentiana* (14 species) are probably restricted to a narrow distribution range owing to their poor dispersal ability [23]. In contrast, *Tripterospermum* (18 species) has the broadest niche and thrives under the warmest and wettest conditions [24]. A total of 279 species of the subtribe Gentianeae are distributed in China, including 231 species in *Gentiana*, 9 species in *Metagentiana*, 9 species in *Kuepferia*, 16 species in *Crawfurdia*, 2 species in *Sinogentiana*, and 16 species in *Tripterospermum* [17]. In the Qinghai-Tibetan plateau, there are about 176 species of subtribe Gentianeae, including Ca. 145 species in *Gentiana*, 3 species in *Metagentiana*, 9 species in *Kuepferia*, 15 species in *Crawfurdia*, 2 species in *Sinogentiana*, and 2 species in *Tripterospermum*.

#### 3.1.2. Subtribe Swertiinae

Subtribe Swertiinae is a larger and more complex group than subtribe Gentianeae. According to Struwe et al., subtribe Swertiinae consists of 579–608 species in 14 genera, including *Bartonia*, *Comastoma*, *Frasera*, *Gentianella*, *Gentianopsis*, *Halenia*, *Jaeschkea*, *Latouchea*, *Lomatogonium*, *Megacodon*, *Obolaria*, *Pterygocalyx*, *Swertia*, and *Veratrilla* [16]. Ho et al. published two new genera, *Lomatogoniopsis* and *Sinoswertia* [25,26]. Therefore, there are 16 genera in the subtribe Swertiinae, of which 13 genera are native to China, including 3 endemic genera. A total of 131 species of subtribe Swertiinae are found in China, including 11 species in *Comastoma*, 9 species in *Gentianella*, 5 species in *Gentianopsis*, 2 species in *Halenia*, 2 species in *Jaeschkea*, 1 species in *Latouchea*, 3 species in *Lomatogoniopsis*, 17 species in *Lomatogonium*, 2 species in *Megacodon*, 1 species in *Sinoswertia*, 76 species in *Swertia*, and 2 species in *Veratrilla* [17]. In the Qinghai-Tibetan plateau, there are about 98 species of subtribe Swertiinae, including 9 species in *Comastoma*, 7 species in *Gentianella*, 4 species in *Gentianopsis*, 2 species in *Halenia*, 2 species in *Jaeschkea*, 3 species in *Lomatogoniopsis*, 15 species in *Lomatogonium*, 1 species in *Megacodon*, 1 species in *Sinoswertia*, 52 species in *Swertia*, and 2 species in *Veratrilla*. Although subtribe Swertiinae can be morphologically divided into two large groups (Rotate and Tubular), molecular phylogeny shows that several groups within subtribe Swertiinae are distinct complex groups [20]. *Swertia* is the main group of subtribe Swertiinae, whereas other related genera, either monophyletic or symphyletic, are derived from *Swertia* [27]. There are significant inconsistencies between the morphological taxonomy and molecular phylogeny among the genera of subtribe Swertiinae. The possible explanation is that morphological taxonomy cannot reflect the true evolutionary history, and the most significant reason may be the plasticity of the morphological characteristics [27,28,29].

### 3.2. Ethnomedicinal Usage of Gentianeae in Tibetan Medicine

**Figure 1:**
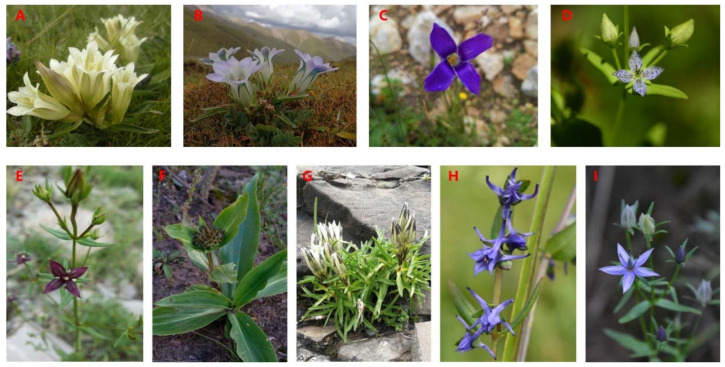
Representative Gentianeae species used in Tibetan medicine ((**A**). Gentiana straminea; (**B**). Gentiana szechenyii; (**C**). Gentianopsis paludosa; (**D**). Sinoswertia tetraptera; (**E**). Swertia mussotii; (**F**). Gentiana crassicaulis; (**G**). Gentiana algida; (**H**). Halenia elliptica; (**I**). Swertia franchetiana).

Gentianeae species are important resources in traditional Chinese medicine and Tibetan medicine [5,30,31]. Approximately 133 species (including varieties) in 17 genera of Gentianeae were used in traditional Chinese medicine, among which *Gentiana* and *Swertia* constituted the largest number of species with 60 and 35 species, respectively, accounting for 71% of the species [32]. According to our investigation, approximately 83 species were used in Tibetan medicine (Table 1), including 3 species of *Comastoma*, 42 species of *Gentiana*, 3 species of *Gentianopsis*, 3 species of *Halenia*, 5 species of *Lomatogonium*, 1 species of *Megacodon*, 1 species of *Sinoswertia*, 24 species of *Swertia* and 1 species of *Veratrilla* (Figure 1). The history of the use of Gentianeae species in Tibetan medicine can be traced back to the 8th century in the book “The Remain of Dunhuang Tibetan Medicine” [33], “Sman-dpyad Zla-bavirgyal-po” [6], and “Dpl Ldn Rgyud Bzhi” [7], in which there are descriptions of the efficacy of Tibetan medicines, such as Bangjian (སྤང་རྒྱན།), Jieji (ཀྱི་ལྕེ།), Dida (ཏིག་ཏ།), and Ganggaqiong (གང་གྰཆུང་།), belonging to Gentianaceae. These classical works of Tibetan medicine not only record the use and usage of the medicine but cover the morphological characteristics and habitats of the plants used in traditional Tibetan medicine.

#### 3.2.1. Bangjian (སྤང་རྒྱན།)

“Bangjian” is the general name of several medicinal plants of *Gentiana* and is a representative and commonly used term in Tibetan medicine. These plants are mainly used for the treatment of respiratory diseases, such as pneumonia, cough, tracheitis, and laryngitis, and fever [5,6,7,8]. According to our investigation, 15 species of *Gentiana* plants were used as the material medica resources of “Bangjian.” In accordance with the classification and nomenclature of Tibetan medicine, the material medica resources of “Bangjian” are classified by flower color. “Shel Gong Shel Phreng” divides “Bangjian” into three types: white (Bangjian·Gabao [སྤང་རྒྱན་དཀར་པོ།]), blue (Bangjian·Wenbao [སྤང་རྒྱན་སྔོན་པོ།]), and black (Bangjian·Nabao [སྤང་རྒྱན་ནག་པོ།]) [8]. “Bee Sngon” (བེེཌྰུརྱ་སྔོན་པོ།) divided “Bangjian” into white flower (Bangjian·Gabao), blue flower (Bangjian·Wenbao), and other colored flowers (Bangjian·Chabao [སྤང་རྒྱན་ཁྲ་པོ།]), among which the white flower was the best [34].

“Bee Sngon” documents that “Bangjian·Gabao grows on grassy slopes. The leaves are small, and the flowers are abundant. The taste is bitter and the effect is to cure the fever epidemic” [34]. “Shel Gong Shel Phreng” records that “Bangjian·Gabao grows in the alpine cold region. The leaves are like Jieji·Gabao (ཀྱི་ལྕེ་དཀར་པོ།) with no stem. Four or five white flowers are clustered with red luster” [8]. “Sgrol Ma Sngo Vbum” (བོད་སྨན་ཚད་ལྡན་འཁྲུངས་དཔེ།) states that “the leaves of Bangjian·Wenbao are like the Bangjian·Gabao. It is bitter in taste and cold-natured” [35]. “Shel Gong Shel Phreng” wrote “Bangjian·Wenbao grows on very wet marsh grassy flats with small leaves and pale blue flowers. The function is consistent with Bangjian·Gabao” [8]. “Shel Gong Shel Phreng” wrote “the flowers of Bangjian·Nabao are dark blue, very conspicuous, and slightly bolder than the Bangjian·Wenbao” [8]. “Bdud Rthi Smn Gyi Vkhrung Dpe” (བདུད་རྩི་སྨན་གྱི་འཁྲུངས་དཔེ་ལེགས་བཤད་ནོར་བུའི་ཕྲེང་མཇེ།) recorded that “the roots of Bangjian·Chabao were light yellow with fibrous roots. The leaves and stems were like Bangjian·Nabao, but without branches. The flowers are variegated and shaped like horns” [36]. “Tibetan medicine” (༄༄།།བོད་སྨན་གྱི་རྣམ་བཤད།) reports that “Bangjian·Chabao may refer to the distinct heterochromatic stripes and spots in the corolla, similar to Bangjian·Nabao, and not easily distinguishable from the Bangjian·Wenbao” [5].

The experience of the usage of “Bangjian” was summarized by Tibetan ancestors. It is extremely complicated to determine the origin of its medicinal materials because of the lack of systematic taxonomic knowledge and detailed description in the classic works of Tibetan medicine [14,37,38]. Herbological studies on the material medica resources of “Bangjian” are, therefore, necessary. After a systematic review and analysis of herbalism and phytotaxonomy in both ancient and modern literature, it is widely believed that *G. szechenyi*i and *G. algida* (including *G. algida* var. *algida* and *G. algida* var. *purdomii*) are the two original plants of “Bangjian·Gabao” [39]. Nevertheless, research on the material medica resources of Bangjian·Nabao, Bangjian·Wenbao, and Bangjian·Chabao is extremely difficult as existing standards in the material medica resources are inconsistent [40,41,42]. Several plants in *Gentiana,* Sect. *Monopodiae,* Ser. *Ornatae* were used as the resources of “Bangjian·Nabao”, “Bangjian·Wenbao”, and “Bangjian·Chabao” (Table 1). These plants are very similar in morphology and have different flowers and colors in different regions, seasons, and habitats. It is impossible for Tibetan doctors and farmers in the field to distinguish medicinal materials based on plant taxonomy; thus, the situation of mixed use arises during production [40,41,43,44]. Although the material medica resources of “Bangjian·Nabao”, “Bangjian·Wenbao”, and “Bangjian·Chabao” are mixed, their effects are similar. These components are mainly used to treat fever and respiratory diseases, and the mixed components may not affect efficacy. It is particularly important to set more reasonable standards and thoroughly study the material medica resources of “Bangjian”.

#### 3.2.2. Jieji (ཀྱི་ལྩེ།)

“Jieji” is a widely used Tibetan medicine. “Shel Gong Shel Phreng” documented that “Jieji” can clear fever of the viscera and gallbladder and can also cure leprosy [8]. It is widely used in the treatment of acute jaundrax hepatitis, rheumatoid arthritis, tonsillitis, leprosy, and urticaria [45]. According to our investigation, 9 species in the *Gentiana* Sect. *Cruciata* were used as the material medica resources of “Jieji.” “Shel Gong Shel Phreng” divided “Jieji” into two types: white flower (Jieji·Gabao [ཀྱི་ལྩེ།་དཀར་པོ།]) and black flower (Jieji·Nabao [ཀྱི་ལྩེ།་ནག་པོ།]) [8].

“Shel Gong Shel Phreng” recorded that “Jieji·Gabao grows in hillside meadows. Its stems are red with thick green leaves that are long and glossy. The flowers are white with many green stripes. The stems are erect with apical flowers like Bangjian” [8]. “Tibetan medicine” considers the abovementioned plant with red stems, white flowers, and green stripes as *G. straminea* and the plant with erect stems and apex flowers is *G. tibetica* or *G. officinalis* [5]. “Bee Sngon” states that “Jieji·Nabao growing in the mountains has morphological characteristics like Jieji·Gabao, with blue-purple flowers and thin leaves” [34]. “Shel Gong Shel Phreng” described that “Jieji·Nabao was like Jieji·Gabao, but the stem and leaves tiled on the ground. The leaves are slightly large and the flowers are white without significant luster” [8]. “Tibetan medicine” considered that the material medica resources of “Jieji·Nabao” are mainly *G. dahurica* and *G.crassicaulis* [5]. Moreover, other material medica resources of Jijie were used in actual production [46]. In the Qinghai Province, Tibetan doctors use *G. hexaphylla* as “Jieji·Gabao”. In Tibet, *M. stylophorus* was used as “Jieji·Gabao·Manba” (ཀྱི་ལྩེ།་དཀར་པོ་དམན་པ) [47]. In addition, *G. waltonii*, *G. robusta*, and *G. siphonantha* were also used as “Jieji” [47].

#### 3.2.3. Dida (ཏིག་ཏ།)

“Dida” is one of the most representative medicines used in Tibetan medicine. “Shel Gong Shel Phreng” recorded that “Dida” has the effect of purging the liver and gallbladder, promoting diuresis, renewing the physique, and stanching bleeding [8]. Currently, “Dida” is widely used in the treatment of acute jaundice hepatitis, viral hepatitis, cholecystitis, urinary tract infections, blood disease, injuries, dysentery, edema, influenza, and other diseases [48]. 

**Figure 2:**
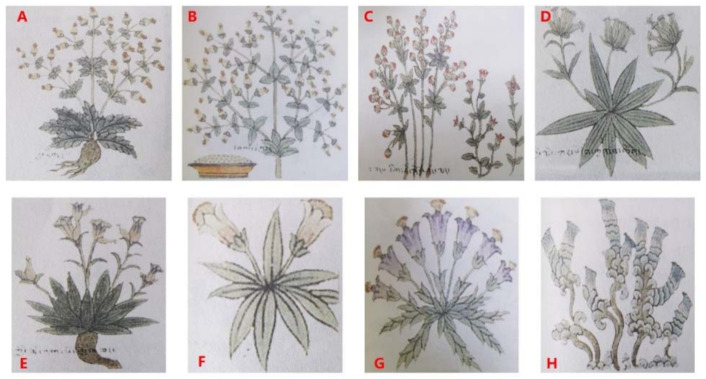
Mantang (སྨན་ཐང་རྒྱལ་འགྲེལ།) of the representative Gentianeae species in Tibetan medicine ((**A**). Jiadi [རྒྱ་ཏིག]; (**B**). Wadi [བལ་ཏིག]; (**C**). Sangdi [ཟངས་ཏིག]; (**D**). Jieji·Gabao [ཀྱི་ལྩེ།་དཀར་པོ།]; (**E**). Jieji·Nabao [ཀྱི་ལྩེ།་ནག་པོ།]; (**F**). Bangjian·Gabao [སྤང་རྒྱན་དཀར་པོ།]; (**G**). Bangjian·Nabao [སྤང་རྒྱན་ནག་པོ།]; (**H**). Ganggaqiong [གང་གྰཆུང་།]) [52].

“Sman-dpyad Zla-bavirgyal-po” divides “Dida” into three categories according to its origin: “Indian Dida” (“Jadi”, རྒྱ་ཏིག), “Nepalese Dida” (“Wadi”, བལ་ཏིག), and “Tibet Dida” (“Wodi”, བོད་ཏིག) [7]. “Shel Gong Shel Phreng” followed the above classification and further divided “Tibet Dida” into six groups: “Songdi” (སུམ་ཏིག), “Saierdi”(གསེར་ཏིག), “Edi” (དངུལ་ཏིག), “Sangdi” (ཟངས་ཏིག), “Jihedi” (ལྕགས་ཏིག), and “Gouerdi”(གུར་ཏིག) [8]. “Shel Gong Shel Phreng” recorded that “Jiadi is like a shrub and the whole plant is shiny. The stem is hollow and knobbly and the epidermis is thin and hard. The leaves are thick and dark green” [8]. It is widely believed that *S. sinensis* is the material medica resource of “Jiadi” [49,50]. *S. sinensis* is mainly distributed in India and Nepal and was recently found to be distributed in China (Dingjie and Jilong conty in Tibet) [51]. “Shel Gong Shel Phreng” documents that “In contrast to Jiadi, the color of Wadi is light and soft and the leaves are slightly yellow, whereas the other characteristics are similar” [8]. According to literature records and textual research, *S. ciliate*, *S. racemosa*, and *C. pedunculatum* are the material medica resources of “Wadi”, among which, the morphological characteristics of *S. ciliate* are more consistent with the picture of “Wadi” in Mantang (སྨན་ཐང་རྒྱལ་འགྲེལ།, Figure 2) [52]. “Tibetan medicine” considers that *C. pedunculatum* was used as “Wadi” in some regions; however, more evidence is needed to confirm these findings [5]. “Shel Gong Shel Phreng” documented that “Songdi grows in rock mountains, grassy slopes, under forests, and rock crevices. The stem is red and the leaves are small. The basal leaves are dense like rosettes with red and yellow flowers” [8]. The literature suggests that the material medica resources of “Songdi” are mainly from *Saxifraga* [5,8,47]. “Shel Gong Shel Phreng” documented that “Saierdi grows in fields and the beach. The stem and leaf are upward extensions with red or yellow flowers. The seeds are yellow and small” [8]. The material medica resources of “Saierdi” are also from *Saxifraga* in the literature [5,8,47]. “Shel Gong Shel Phreng” documents that the “stems and leaves of Edi are quite long and the flowers are white” [8]. Currently, the doctors in Tibet mainly use species from *Parnassia* as “Edi”, whereas those in Qinghai mainly use species in *Cerastium* as “Edi” [5,47]. However, their morphological characteristics are not consistent with the above description. The material medica resources of “Edi” remain to be further characterized. According to “Shel Gong Shel Phreng”, “Sangdi” is characterized by “tufted and red stems, clustered leaves, and slightly hairy, red and yellow flowers” [8]. This is similar to the genus *Saxifraga*, but according to the actual use situation in various regions, “Sangdi” is considered a variant of *Swertia*, which gradually formed in the long history of use under the influence of the distribution of resource species in different regions [5,47]. As recorded in “Shel Gong Shel Phreng”, the stems of “Jihedi” are like iron chopsticks; the leaves are green, the flowers are light blue or blue, and the fruits are similar to flax seeds [8]. The literature holds that the material medica resources of “Jihedi” are mainly species of *Halenia* and *Gentiuanopsis* [5,47,49,53]. Other *Swertia* and *Lomatogonium* plants recorded in Tibetan Medicine as “Jihedi” may be regional substitutes. “Shel Gong Shel Phreng” records that “Gouerdi grows in the shady area of green slopes. The leaves are thick, basal, and with beady, silver spots. The flowers are pale-yellow” [8]. These likely appear to be plants of *Saxifraga* or *Parnassia* [5]. However, some literature considers *S. nigroglandulifera* and *Mentha haplocalyx* as “Gouerdi”, which need to be further confirmed [47,54].

#### 3.2.4. Others

Many other Gentianaceae plants are also used in Tibetan medicine. “Ganggaqiong” (གང་ག་ཆུང་།), “Shel Gong Shel Phreng” recorded that “It tastes bitter, has heat-clearing and detoxifying effects, and cures blood diseases and Chiba disease. It grows in mountains at high altitudes and the roots are similar to tendons. The plants are fluffy and messy with overlapping leaves, which are similar to eight-corner pagodas. The flowers are in white and urceolate in shape” [8]. Doctors in Tibet and Qinghai (Yushu) mainly use *G. urnula* as “Ganggaqiong” [5]. Furthermore, in some regions of Tibet, *G. phyllocalyx* was used as “Ganggaqiong·Manba” (གང་ག་ཆུང་དམན་བ།) [47]. “Shel Gong Shel Phreng” documents that “Saibao·Guzhui (སེར་པོ་རྒུ་དྲུས།) can detoxify and cure sores. It can be divided into two types and the top grade grows in the mountains. The stems are very long and the leaves are thin and smooth similar to those of Jieji·Gabao. The flowers are pale yellow” [8]. Doctors in Tibet mainly use *S. multicaulis* and *S. kingie* as “Saibao·Guzhi” [47].

In addition, *G. aristata* was used as a substitute for “Wengbu” (སྔོན་བུ།); *G. spathulifolia* was used as “Aolamu” (སྔོ་ལྟ་མོ།); *G. crassuloides* was used as “Ebu·youyou” (སྔོན་བུ་ཡོལ་ཡོལ།); *G. rigescens* was used as “Axue·Dida” (ཨ་ཉོག་ཏིག་ཏ།); *S. marginata* and *S. bifolia* were used as “Daiwa” (དེ་བ།) [47].

#### 3.2.5. Formulas

The clinical application of Tibetan medicine is mainly as formulas and single herb preparations are very few. Formulas can contain more than 100 drugs, and those with more than 10 drugs are usually common. Similar to traditional Chinese medicine formulas, Tibetan medicine formulas also employ the “Monarch, Minister, Assistant, and Guide” methods. Some drugs must be processed before use to eliminate and reduce their toxicity and improve efficacy. Decoctions, powders, and water pills are the most common dosage forms, of which powders and water pills are the most widely used in a clinical setting [4,55].

According to our statistics, about 56 formulas containing “Jieji”, 53 formulas containing “Bangjian”, 160 formulas containing “Dida”, and 14 formulas containing “Ganggaqiong” were used (Supplement Table S1). “Jieji” has mostly been used in formulas owing to its effect of clearing heat toxins, removing food stagnation, dispelling wind, and eliminating dampness, and purging the liver and gallbladder. “Bangjian” has mostly been used in formulas owing to its effect of clearing lung heat. “Dida” has been mostly used in formulas because of its heat-toxin clearing effect and purging the liver and gallbladder. “Ganggaqiong” has mostly been used in formulas owing to its effect of clearing heat toxins.

### 3.3. Phytochemistry of Gentianeae

To date, most phytochemical studies have mainly focused on the *Gentiana* and *Swertia* genera. Nearly 600 metabolites were identified from the *Gentiana* genus [56,57,58] and more than 400 metabolites were identified from the *Swertia* genus [59,60,61,62]. The primary bioactive components isolated from the two genera include iridoids, xanthones, flavonoids, and triterpenoids, of which a few are alkaloids, lignans, and phenolic compounds.

#### 3.3.1. Iridoids

As the main chemical constituents, iridoids are widely distributed within the *Gentiana* and *Swertia* genera [16]. Iridoids can be divided into six groups including loganic acid, secologanic acid, morroniside, sweroside, swertiamarin, and gentiopicroside derivatives based on the classic mevalonic acid pathway [63]. Loganic acid derivatives mostly belong to carbocyclic iridoids and most are esters of loganic acid [63]. To date, more than 40 loganic acid derivatives were isolated from *Gentiana* [56], whereas only 10 were isolated from *Swertia* [61]. Secologanic acid derivatives are mainly derived from the split C7–C8 bond of carbocyclic iridoids. About 19 secologanic acid derivatives were obtained from *Gentiana* [56,63] and only two (vogelioside and nervoside) from *Swertia* [16]. Morroniside derivatives were not the major iridoids in both genera. To date, only 13 morroniside derivatives were isolated from *Gentiana* and none from *Swertia* [64]. Sweroside, swertiamarin, and gentiopicroside derivatives were not only the major compounds in *Gentiana* and *Swertia* but are also widely distributed [16,56,62]. In the biosynthetic pathway, sweroside, swertiamarin, and gentiopicroside are the precursors of the latter [63]. Gentiopicroside derivatives were characterized in the existence of double bond in C-5 and C-6, whereas sweroside derivatives without the double bond and swertiamarin derivatives with hydroxyl at C-5, 22, 14, and 25 of sweroside, swertiamarin, and gentiopicroside derivatives was obtained, respectively, in *Gentiana* while were obtained respectively in *Swertia* [56,61].

#### 3.3.2. Xanthones

Xanthones are rather rare among higher plants and are found almost exclusively in Gentianaceae, Guttiferae, Moraceae, Clusiaceae, and Polygalaceae [16,65]. Unlike iridoids, xanthones are not present in all plant species that have been investigated in the Gentianaceae family [16]. Xanthones isolated from natural sources are classified into six groups, namely, simple xanthones, xanthone glycosides, prenylated xanthones, xanthonolignoids, bisxanthones, and miscellaneous xanthones [66]. Only simple xanthones and xanthone glycosides were reported in Gentianaceae [16,56,58,61]. In Gentianeae, 8-substituted or 2- and 4-substituted xanthones are predominant [16]. Xanthone glycosides were predominantly reported in the Gentianaceae and Polygalaceae families as C- or O-glycosides [67,68]. Mangiferin is the most common C-glycoside xanthone in *Gentiana* and *Swertia* [56,61]. Besides, some Mangiferin derivatives such as neomangiferin and mangiferin-6-O-β-d-glucopyranoside have also been obtained from the two genera [69,70]. The first O-glycoside xanthone, norswertianin-1-O-glucosyl-3-O-glucoside, was isolated from *S. perennis* [71]. Other xanthone O-glycosides such as gentiacauloside from *G. acaulis* [72], gentioside from *G. marcailhouana* [73], and swertianolin from *S. chirayita* [74] have also been isolated.

#### 3.3.3. Flavonoids

The flavonoids in Gentianeae mainly exist as free or in the glycosidic forms. A total of 58 and 12 flavonoids were obtained from *Gentiana* and *Swertia*, receptively [56,61,75,76,77]. The majority of the glycosylated flavonoids are isoorientin and isovitexin derivatives, which implies that both might be the key compounds in the biosynthetic pathway of flavonoids in Gentianeae. (2*E*)-1-(2-Hydroxyphenyl)-3-{5′-[3-(2-hydroxyphenyl)-3-oxopropyl]-2′,6-bis[(3-methylbut-2-en-1-yl)oxy]-biphenyl-3-yl}prop-2-en-1-one isolated from the bark of *G. lutea*, is the only flavonoid dimer reported from Gentianaceae [78], whereas swertifrancheside from *S. franchetiana* was the first flavone-xanthone dimer to be isolated [79].

#### 3.3.4. Triterpenoids

Gentianeae is also rich in triterpenoids. A total of 64 triterpenoids were isolated from *Gentiana* and classified, and 33 were isolated from *Swertia* [56,61]. The carbon frameworks of triterpenoids in *Gentiana* include dammarane, oleanane, ursane, lupane, hopane, chiratane, sterol, and squalene skeletons. Carbon skeletons of triterpenoids in *Swertia* consist of oleanane, ursane, taraxerane, lupane, hopene, isohopane, gammacerane, swertane, chiratane, and lanostane skeletons. Oleanane-type triterpenoids are the most substantial. Oleanolic acid was once recommended as a marker by some researchers to assess the quality of “Dida”, owing to its relatively high concentrations [51].

### 3.4. Pharmacology of Gentianeae

The bioactivities of plants from Gentianeae include hepatic protection, upper respiratory tract protection, joint and bone protection, glucose regulation, antibacterial, antioxidant, anticancer, and antiviral effects (Table 2).

#### 3.4.1. Hepatic Protection

“Dida” is the most common medicinal material for the treatment of hepatitis, especially icteric hepatitis [48]. The mechanism mainly involves restoring abnormal physiological characteristics and biochemical indexes of liver, improving antioxidants levels and lipid peroxidation, and promoting blood supply to the liver tissue [80,81,82,83]. Plants from “Jieji” also showed similar hepatoprotective effects [84]. Gentiopicroside and Swertiamarin, the main iridoid in “Jieji” plants, can significantly reduce the abnormal biochemical indexes of liver and increase the antioxidants and lipid peroxidation levels [85,86,87]. 

#### 3.4.2. Protection from Upper Respiratory Tract Infections

“Bangjian” is widely used in the treatment of upper respiratory tract infections. More than 60% of the formulas with “Bangjian” as the primary drug are clinically used for the treatment of respiratory diseases such as bronchitis, emphysema, acute and chronic pharyngitis, cough and asthma, and hoarseness (Table S1). Pharmacological studies reveal that the mechanism involves enhancing p-ERK expression [88] and antioxidant enzymes activities [89], while inhibiting TNF-α, IL-10 and TGF-β1 expression [88,90,91]. 

#### 3.4.3. Joint and Bone Protection

“Jieji” can effectively protect joints and bones. The extracts of *G. straminea*, *G. macrophylla* and *Sinogentiana striata* were proved to alleviate adjuvant arthritis, synovial inflammation and rheumatoid arthritis by effect on different targets and pathways [92,93,94]. Gentiopicroside and swertiamarin also played an important role in joint and bone protection through inactivation of JNK and NF-кB signaling pathways or interfere with the release of inflammatory factors [95,96,97]. 

#### 3.4.4. Glucose Regulation

Many compounds from Gentianeae, especially xanthones and iridoid derivatives, were reported to have glucose-regulating effects [98,99,100]. Demethylbellidifolin can stimulate the GLP-1 receptor in a concentration-dependent manner and reduce blood glucose levels [101]. Bellidifolin was observed to reduce blood glucose levels, indicating its hypoglycemic effect [102]. Swertiamarin can significantly reduce the levels of fasting blood glucose, HbA1c, TC, tTG, and LDL; increase the levels of hemoglobin, insulin, TP, and HDL; and significantly promote the regeneration of pancreatic islet tissue in diabetic rats [103]. Swertiamarin may regulate the expression of related genes in the liver and adipose tissue by targeting PPARγ [104].

#### 3.4.5. Antibacterial Effects

The extract of *G. veitchiorum* is effective against MRSA and MSSA infections [105]. The ethyl acetate extracts of *G. algida* showed antibacterial activity against *Escherichia coli, S. aureus*, *Streptococcus pneumoniae*, *Pseudomonas aeruginosa*, and *Bacillus licheniformis* [106]. The volatile oil of *S. mussotii* exerts antibacterial activity against *S. aureus*, *E. coli*, and *Salmonella typhi* [107]. l,8-Dihydroxy-3,7-dixanthone from *S. mussotii* was found to have antibacterial effect on *Clostridium*, *P. aeruginosa*, *B. megalosus*, *S. aureus*, *Ocardiococcus*, *E. coli*, and *Proteus*, especially *E. coli* [108].

#### 3.4.6. Antioxidant Effects

The extract of *S. chirata*, *S. davidii* and *G. urnula* was found to have the high free-radical scavenging capacity, which is associated with phenolic compounds and synergistic effects with xanthones and glycosides [109]. Xanthones are the major compounds in *Swertia* and this species may, therefore, prove useful in scavenging OH·and O_2_. Studies report an increase in radical-scavenging activity when the substituents are present in the ortho-position [110]. 

#### 3.4.7. Anti-Inflammatory Effects

The extracts of *G. macrophylla*, *G. straminea* and *S. chirayita* all possessed significant anti-inflammatory activities [92,111,112]. Numerus compounds isolated from Gentianeae plants play an important role in anti-inflammatory activity, such as gentiopicroside, swertiamarin 1,5-dihydroxy-3,8-dimethoxy xanthone and Bellidifolin [113,114,115,116]. 

#### 3.4.8. Anticancer

The triterpenoids (24-hydroxyoleanolic acid, 1a,2a,3b,24-tetrahydroxyolean-12-en-28-oicacid, 2a-hydroxyursolicacid) isolated from *G. aristata* showed low cytotoxic activities against HL-60 cells [117]. Six compounds (3-antene, arborinone, boehmerol, carotenoside, 3β-acetoxy-28-hydroxy-12-ene-urthulane and swertisin) from *G. algida* showed different degrees of antitumor activity in HeLa cells [106]. Waltonitone isolated from *G. waltonii* induces tumor cell cycle arrest through regulating Akt and ERK1/2 pathways, thereby inhibiting tumor cell growth [118]. Swertiamarin had certain inhibitory and pro-apoptotic effects on hepatocellular carcinoma cells *in vitro* [119]. Swertiamarin and mangiferin from *S. davidii* are the main active substances that inhibit growth and induce apoptosis in HepG2 human liver cancer cells [120].

#### 3.4.9. Antiviral Effects

Both aqueous and alcohol extracts of *G. macropylana* significantly inhibit influenza A and B [121,122]. The extracts of *G. veitchiorum* showed obvious inhibitory effects on RSV both *in vivo* and *in vitro* [123]. Lots of compounds isolated from Gentianeae had significantly anti-HBV and anti-HIV activity [79,124,125,126,127]. 

## 4. Limitation 

This review paper is based on the author’s own analysis and summary of the literature. Although the author tries to keep objective in the analysis process, it is still highly subjective, thus all the findings are based on personal views. This review paper only covers the research results published in mainstream journals from 2000 to 2021, and it is inevitable to overlook some of them. Therefore, readers need to understand the limitations of this review paper in terms of time and sources. There have been previous reviews on the chemical constituents and pharmacological activities of *Gentiana* and *Swertia* [56,60]. Nevertheless, in this review, we mainly focused on the related studies of Gentianeae plants used in Tibetan medicine, especially ethnomedicinal usage. Moreover, we also included the latest research results.

## 5. Conclusions

Due to its complete theoretical system and remarkable therapeutic effect, Tibetan medicine has attracted increasing attention from researchers. However, proper research on Tibetan medicine is scarce. In this study, we employ Gentianeae as a case, aiming to provide good inspiration for the follow-up research in Tibetan medicine.

Species from Gentianeae have been widely and long used as Tibetan medicine. Many species are good sources for chemical and pharmaceutical research owing to the presence of high levels of iridoids, flavonoids, and triterpenoids. However, the ethnomedicinal usage and the phytochemical and pharmacological properties of Gentianeae in Tibetan medicine were not well summarized. In view of this, we systematically summarize the ethnomedicinal usage and the phytochemical and pharmacological properties of Gentianeae in Tibetan medicine.

Although various classes of compounds were identified and their pharmacological activities investigated, systematic studies are lacking for numerous species. Thus, there is the likelihood of the presence of undiscovered compounds. Therefore, phytochemical profiling, bioactivity screening, biosynthetic pathway elucidation, and structure-activity relationship studies should be continued, as these findings could provide a more reasonable foundation for use of Gentianeae species and the maximization of their desired therapeutic benefits.

## Figures and Tables

**Table 1:** Gentianeae species used in Tibetan medicine.

No.	Species	Genus	Tribes	Tibetan Medicine Name
1	*G. algida* var. *purdomii*	*Gentiana*	Subtribe Gentianeae	Bangjian (སྤང་རྒྱན།)
2	*G. acaulis*			
3	*G. algida*			
4	*G. altorum*			
5	*G. arethusae* var. *delicatula*			
6	*G. atuntsiensis*			
7	*G. filistyla*			
8	*G. futtereri*			
9	*G. lawrencei* var. *farreri*			
10	*G. nubigena*			
11	*G. obconica*			
12	*G. ornata*			
13	*G. purdomii*			
14	*G. sinoornata* var. *gloriosa*			
15	*G. stipitata*			
16	*G. szechenyii*			
17	*G. veitchiorum*			
18	*G. yunnanensis*			
19	*G. cephalantha*	*Gentiana*	Subtribe Gentianeae	Jieji (ཀྱི་ལྕེ།)
20	*G. crassicaulis*			
21	*G. dahurica*			
22	*G. erectosepala*			
23	*G. hexaphylla*			
24	*G. macrophylla*			
25	*G. officinalis*			
26	*G. rhodantha*			
27	*G. robusta*			
28	*G. siphonantha*			
29	*G. straminea*			
30	*G. tibetica*			
31	*G. waltonii*			
32	*M. stylophorus*	*Megacodon*	Subtribe Swertiinae	Jieji (ཀྱི་ལྕེ།)
33	*G. pseudosquarrosa*	*Gentiana*	Subtribe Gentianeae	Ganggaqiong (གང་གྰཆུང་།)
34	*G. phyllocalyx*			
35	*G. urnula*			
36	*G. wardii*			
37	*G. aristata*	*Gentiana*	Subtribe Gentianeae	Wengbu (སྔོན་བུ།)
38	*G. capitata*			
39	*G. haynaldii*			
40	*G. crassuloides*	*Gentiana*	Subtribe Gentianeae	Ebu·youyou (སྔོན་བུ་ཡོལ་ཡོལ།)
41	*G. lhassica*	*Gentiana*	Subtribe Gentianeae	Edaiwa (སྡོ་དེ་བ།)
42	*G. rigescens*	*Gentiana*	Subtribe Gentianeae	Axue·Dida (ཨ་ཉོག་ཏིག་ཏ།)
43	*G. spathulifolia*	*Gentiana*	Subtribe Gentianeae	Aolamu (སྔོ་ལྟ་མོ།)
44	*S. angustifolia* var. *pulchella*	*Swertia*	Subtribe Swertiinae	Dida (ཏིག་ཏ།)
45	*S. bimaculata*			
46	*S. ciliata*			
47	*S. cincta*			
48	*S. dichotoma*			
49	*S. erythrosticta*			
50	*S. franchetiana*			
51	*S. leducii*			
52	*S. mussotii*			
53	*S. nervosa*			
54	*S. paniculata*			
55	*S. punicea*			
56	*S. racemosa*			
57	*S. speciosa*			
58	*Swertia wardii*			
59	*S. younghusbandii*			
60	*S. yunnanensis*			
61	*G. barbata*	*Gentianopsis*	Subtribe Swertiinae	Dida (ཏིག་ཏ།)
62	*G. grandis*			
63	*G. paludosa*			
64	*H. elliptica*	*Halenia*	Subtribe Swertiinae	Dida (ཏིག་ཏ།)
65	*H. corniculata*			
66	*H. elliptica* var. *grandiflora*			
67	*L. forrestii* var. *bonatianum*	*Lomatogonium*	Subtribe Swertiinae	Dida (ཏིག་ཏ།)
68	*L. perenne*			
69	*L. forrestii*			
70	*L. macranthum*			
71	*L. oreocharis*			
72	*S. tetraptera*	*Sinoswertia*	Subtribe Swertiinae	Dida (ཏིག་ཏ།)
73	*S. bifolia*	*Swertia*	Subtribe Swertiinae	Daiwa (དེ་བ།)
74	*S. marginata*			
75	*S. wolfgangiana*			
76	*S. atroviolacea*	*Swertia*	Subtribe Swertiinae	Saibo·Guizhui (སེར་པོ་རྒུ་དྲུས།)
77	*S. elata*			
78	*S. kingii*			
79	*S. multicaulis*			
80	*C. pedunculatum*	*Comastoma*	Subtribe Swertiinae	Jiadi·Jiazha (ལྕགས་ཏིག་ལྕགས་སྦྲག)
81	*C. traillianum*			
82	*C. pulmonarium*			
83	*V. baillonii Franch.*	*Veratrilla*	Subtribe Swertiinae	Bae·Sebao (དཔའ་བོ་སེར་པོ)

**Table 2:** Pharmacological activity of Gentianeae.

Pharmacological Activity	Analytes	Methods	Models	Effects	Reference
**Hepatic protection**	Ethanolic extract of *S. chirata*	Paracetamol	Swiss albino mice	GPT, GOT, ALP and bilirubin↓, LPO↑, SOD, CAT, GSH and GPx↑	[80]
	Ethanolic extracts of *S. mussotii*	CCl_4_	Wistar rats	ALT, AST, TBIL and TBA↓	[81]
	Ethanolic extract of *S. mussotii*	BCG and LPS	Kunming mice	ALT and AST↓	[82]
	Ethanol extract of *S. mussotii*	CCl_4_	Wistar rats	GPT↓, NO↑	[83]
	Aqueous extracts of *G. straminea*	CCl_4_	Kunming mice	ALT and TNF-α↓, IL-10↑	[84]
	Gentiopicroside	CCl_4_	Kunming mice	ALT and AST↓, GSH-Px↑, bilirubin↓	[85]
	Swertiamarin	CCl_4_	SD rats	ALT, AST, ALP↓, MDA↓, SOD, GPx and GSH↑	[86]
	Swertiamarin	D-GalN	Wistar rats	SOD, CAT and GSH↑, MDA↓	[87]
**Protection from upper respiratory tract infections**	Aqueous extract of *G. veitchiorum*	LPS	SD rats	TNF-α↓, p-ERK↑	[88]
	Aqueous extract of *G. veitchiorum*	NH_3_	Kunming mice	SOD↑, MDA↓	[89]
	Ethanol extract of *G. veitchiorum*	NH_3_	Kunming mice	SOD↑, TNF-α and IL-10↓	[90]
	Aqueous extract of *G. veitchiorum*	Ovalbumin	BABL/c mice	TGF-β1↓	[91]
**Joint and bone protection**	Ethanol extracts of *G. macrophylla*	Bovine Type II Collagen	SD rats	INF-γ, anti-cyclic citrullinafer peptide antibody and TNF-α↓, IL-4↑	[92]
	Ethyl acetate extract of *S. striata*	Xylene	SD rats	PGE2 and NO↓	[93]
	Ethanol extracts of *G. macrophylla*	LPS	Kunming mice	IL-1β, IL-6, and TNF-α↓, iNOS and COX-2↓	[94]
	Gentiopicroside	Osteoclast	SD rats	NF-κB p65↓	[95]
	Gentiopicroside	IL-1β	Rat chondrocytes	p38, ERK and JNK↓, PGE2 and COX-2↓	[96]
	Swertiamarin	IL-1β	fibroblast synovial cells	p38↓, COX-2 and PEG2↓	[97]
**Glucose regulation**	Swerchirin	Glucose	Albino rats	blood sugar↓	[98]
	Hexane fraction of *S. chirayita*	Glucose	Albino rats	blood sugar↓, plasma IRI↑	[99]
	Methanolic and aqueous extract of *S. chirayita*	Starch	α-amylase (*in vitro*)	α-amylase↓	[100]
	Demethylbellidifolin		HEK293 cell	GLP-1↑	[101]
	Bellidifolin	Streptozotocin	BABL/c mice	blood glucose↓	[102]
	Swertiamarin	Streptozotocin	Wistar rats	blood glucose, HbA1c, TC, TG and LDL↓, hemoglobin, plasma insulin, TP, body weight and HDL↑	[103]
	Swertiamarin	Streptozotocin	NIDDM rat	G6Pase and HMG-CoA reductase↑, PEPCK, GK, Glut 2, PPAR-γ, leptin, adiponectin, LPL, SREBP-1c, and Glut 4↑	[104]
**Antibacterial effects**	Extract of G. veitchiorum	MRSA	Kunming mice	MRSA and MSSA↓	[105]
	Ethyl acetate extracts of *G. algida*		*in vitro*	*E. coli*, *S. aureus*, *S. pneumoniae*, *P. aeruginosa*, and *B. li-cheniformis*↓	[106]
	Volatile oil of *S. mussotii*		*in vitro*	*S. aureus*, *E. coli*, and *S. typhi*↓	[107]
	l, 8-Dihydroxy-3,7-dixanthone		*in vitro*	*Clostridium*, *P. aeruginosa*, *B. megalosus*, *S. aureus*, *Ocardiococcus*, *E. coli* and *Proteus*↓	[108]
**Antioxidant effects**	Ethanol extract of *S. davidii*		*in vitro*	DPPH and ABTS↓	[109]
	Ethanol extract of *G. urnula*		*in vitro*	DPPH, O2, OH↓	[110]
**Anti-inflammatory effects**	Extracts of *G. macrophylla* and *G. straminea*	LPS		p65, p50 and NF-κB↓	[111]
	Ethanol extract of *G. macrophylla*	Bovine Type II Collagen	SD rats	INF-γ, anti-cyclic citrullinafer peptide antibody and TNF-α↓, IL-4↑	[92]
	Aqueous extract of *S. chirayita*	FCA	Swiss albino mice	TNF-α, IL-1β, IL-6, and IFN-γ↓, IL-10↑	[112]
	Gentiopicroside	LPS	RAW 264.7	NO, PGE2 and IL-6↓	[113]
	Gentiopicroside	xylene	Kunming mice	TNF-α, IL-1β, IL-6, iNOS and COX-2↓	[113]
	Gentiopicroside	Dextran sulfate sodium	ICR mice	TNF-α, IL-1β, IL-6, iNOS and COX-2↓	[114]
	Swertiamarin	FCA	SD rats	IL1, TNF, IL-6, MMPs, iNOS, PGE2, PPARc and COX-2↓, IL-10 and IL-4↑, NF-κB, p65, p-IκBα, p-JAK2 and p-STAT3↓	[115]
	Bellidifolin	LPS	RAW 264.7	COX-2, Akt, IKK-β, MAPK and NF-κB↓	[116]
**Anticancer**	24-hydroxyoleanolic acid, 1a,2a,3b,24-tetrahydroxyolean-12-en-28-oicacid, 2a-hydroxyursolic acid		HL-60	↓	[117]
	3-antene, arborinone, boehmerol, carotenoside, 3β -acetoxy-28-hydroxy-12-ene-urthulane and swertisin		HeLa	↓	[106]
	Waltonitone		bel-7402, PANC-1, BXPC-3, and HeLa	↓	[118]
	Swertiamarin		HepG2	↓	[119]
	Swertiamarin and mangiferin		HepG2	↓	[120]
**Antiviral effects**	Ethanol extracts of *G. macropylana*	Influenza A and B	ICR rats	↓	[121,122]
	Aqueous, n-butanol, ethyl acetate, and chloroform extracts of *G. veitchiorum*	RSV	Kunming mice	↓	[123]
	1,8-dihydroxy-3,5dimethoxy-xanthone, norswertianolin, luteolin neolancerin, and isovitexin	HBV	HepG2.2.15	↓	[124]
	Swermacrolactones A-C	HBV	HepG2.2.15	↓	[125]
	(+)-dehydrodiconiferyl alcohol and dehydrozingerone	HBV	HepG2.2.15	↓	[126]

## Supplementary Materials

The following are available online at https://www.mdpi.com/article/10.3390/plants10112383/s1, Table S1: Formulas containing Gentianeae plants in Tibetan medicine.

## Abbreviations

Akt	protein kinase B
ALP	alkaline phosphatase
ALT	alanine transaminase
AST	aspartate transaminase
BCG	Bacillus Calmette Guerin vaccine
CAT	catalase
COX-2	cyclooxygenase 2
D-GalN	d-galactosamine
DPPH	2,2-Diphenyl-1-picrylhydrazyl
ERK	extracellular regulated protein kinase
ERK1/2	extracellular regulated protein kinase 1/2
G6Pase	glucose-6-phosphatase
GK	glucokinase
GLP-1	glucagon-like peptide-1
Glut2	glucose transporter type 2
Glut4	glucose transporter type 4
GOT	glutamate oxaloacetate transaminase
GPT	glutamate pyruvate transaminase
GPx	glutathione peroxidase
GSH	glutathione
HbA1c	glycosylated hemoglobin
HBV	hepatitis B virus
HCG-CoA	hydroxymethylglutaryl-coenzyme A
HDL	high-density lipoprotein
HIV	human immunodeficiency virus
IKK-β	κB kinase-β
IL-10	interleukin 10
IL-1β	interleukin 1β
IL-4	interleukin 4
IL-6	interleukin 6
INF-γ	Th1-type cytokine interferon γ
iNOS	inducible nitric oxide synthase
ITS	internal transcribed spacer
JAK2/STAT3	janus kinases 2/signal transducer and activator of transcription 3
JNK	Janus kinase
LDL	low density lipoprotein
LPO	lipid peroxide
LPS	lipopolysaccharide
MAPK	mitogen-activated protein kinase
*mat*K	maturase K
MDA	malondialdehyde
MRSA	methicillin-resistant Staphylococcus aureus
MSSA	methicillin-sensitive Staphylococcus aureus
NF-кB	nuclear factor kappa-B
NO	nitric oxide
P38	P38 mitogen-activated protein kinase
PEPCK	phosphoenolpyruvate carboxykinase
p-ERK	phosphorylated extracellular regulated protein kinase
PGE2	prostaglandin E2
PPARγ	peroxisome proliferators-activated receptors γ
RSV	respiratory syncytial virus
SOD	superoxide dismutase
TBA	total bile acid
TBIL	total bilirubin
TC	total cholesterol
TG	thyroglobulin
TGF-β1	transforming growth factor-β1
TNF-α	tumor necrosis factor-α
TP	total protein
*trn*L	transfer RNA L
