# Supplementary material for: A Review on the Ethnomedicinal Usage, Phytochemistry, and Pharmacological Properties of Gentianeae (Gentianaceae) in Tibetan Medicine
Source: Plants (Basel). 2021 Nov 5;10(11):2383. doi: 10.3390/plants10112383 (PMC8620629; doi:10.3390/plants10112383)
Supplement: Supplementary file 1 [file plants-10-02383-s001.zip › plants-1433798-supplementary.pdf]

A Review on the Ethnomedicinal Usage, Phytochemistry, and Pharmacological Properties of Gentianeae (Gentianaceae) in Tibetan Medicine

Xiaofeng Chi[1,2], Faqi, Zhang[1,2], Qingbo Gao[1,2], Rui Xing[1,2], Shilong Chen[1,2]*

[1] Northwest Institute of Plateau Biology, Chinese Academy of Sciences, Xining 810008, China
[2] Qinghai Provincial Key Laboratory of Crop Molecular Breeding, Xining 810008, China
* Correspondence: slchen@nwipb.cas.cn (S.L. Chen)

Supplement Materials

Table S1. Formulas containing Gentianeae plants in Tibetan medicine.

No.	Formulas (Chinese name)	Formulas (Tibetan name)	Containing herbs	Attending functions
1	A Zha He Ming San	A Zha He Ni Da Song Jiao Er	Dida	Heat-clearing and detoxicating
2	Ba Sa Mu La Long	Ba Sa Mu La Long	Dida	Tonifying the kidney
3	Ba Wei Da Tang San	Tang Qing Jie Ba	Dida	Clearing heat
4	Ba Wei Ding Xiang San	Le Xi Jie Ba	Bangjian	Clear and nourish throat
5	Ba Wei Fei Qin San	Xia Bu Er Jie Ba	Dida	Clearing heat
6	Ba Wei Ling Gai San	Mu Tuo Le Jie Ba	Dida	Relieve pain
7	Ba Wei Tu Er Cao San	Hong Lian Jie Ba	Dida	Heat-clearing and detoxicating
8	Ba Wei Xiao Ye Du Juan San	Da Li Jiao Jie	Dida	Stretch the muscles and smooth the vein
9	Ba Wei Zhang Ya Cai San	Dou Da Jie Ba	Dida	Clearing heat and benefiting gallbladder
10	Ba Zhu San	Zuo Wo Jie Ba	Dida	Heat-clearing and detoxicating
11	Bang Cha He San	Bang Cha He	Bangjian	Clearing heat and expectoration
12	Chu Han Da Pei Wu	Zhang Jiao Er Qing Mao	Jieji, Dida, Bangjian	Detoxification
13	Da Hei Wan	Ri Na He Qing Mao	Jieji, Dida	Detoxification
14	Da Hong Wan	Ma Er Qing Ri Bu	Jieji, Bangjian	Clear and nourish throat
15	Da Jian Tang San	Dui Tang Qing Mao	Dida	Heal a wound
16	Da Tie Wan	Jiu He Ri Qing Mao	Dida, Ganggaqiong	Heat-clearing and detoxicating
17	Da Wen Bu San	Mai Nian Ca Na He Qing Mao	Dida	Clearing heat
18	Da Xiang Hong Ga Mu Xie	Lang Qing Ga Mu Xie Ma Er Bao	Dida	Dispersing stagnated liver
19	Da Yue Jing San	Da Xie Qing Mao	Dida	Clearing heat
20	De Ma Er Shi San Wei Niu Huang San	De Ma Er Ge Wang Ju Song	Dida	Heat-clearing and detoxicating
21	Duo Pei San	Mang Jiao Er	Dida	Heat-clearing and detoxicating
22	Duo Pei Zhang Ya Cai	Dou Da Mang Jiao Er	Dida	Heat-clearing and detoxicating
23	Er Shi Er Wei Dan Tang San	Chi Tang Ni Er Ni	Dida, Bangjian	Cholagogic
24	Er Shi San Wei Mao Ru San	Sang Dang Ni Er Song	Jieji	Dispelling wind and eliminating dampness
25	Er Shi Si Wei He Zi San	A Ri Ni Er Yu	Dida	Tonifying the kidney
26	Er Shi Wei Mu Xiang San	Ri Da Ni Xi	Ganggaqiong	Insecticidal
27	Er Shi Wu Wei Bing Pian San	Ga Bu Er Ni Er A	Dida	Heat-clearing and detoxicating
28	Er Shi Wu Wei Cao Wu San	Man Qing Ni Er A	Dida	Relieve pain
29	Er Shi Wu Wei Da Tang San	Tang Qing Ni Er A	Dida, Ganggaqiong	Dispelling wind and eliminating dampness
30	Er Shi Wu Wei Da Xiang Hua San	Lang Qing Mai Duo Ni Er A	Dida	Detumescence
31	Er Shi Wu Wei Ding Xiang San	Le Xi Ni Er A	Dida	Sedative
32	Er Shi Wu Wei Duan Jie San	Jia Le Er Ni Er A	Ganggaqiong	Hemostatic
33	Er Shi Wu Wei Han Shui Shi San	Gui Xi Ni Er A	Dida	Clearing stomach-heat
34	Er Shi Wu Wei Hu Fei San	Wa Luo Ni Er A	Dida, Bangjian	Clearing lung-heat
35	Er Shi Wu Wei Jin Yao Cao San	Ya Ji Ni Er A	Jieji	Heat-clearing and detoxicating
36	Er Shi Wu Wei Ju Tang San	De Le Tang Ni Er A	Dida	Heat-clearing and detoxicating
37	Er Shi Wu Wei Liu Gan Tang San	Qia Mu Tang Ni Er A	Dida, Ganggaqiong	Heat-clearing and detoxicating
38	Er Shi Wu Wei Long Dan San	Bang Jian Ni Er A	Bangjian, Ganggaqiong	Heat-clearing and detoxicating
39	Er Shi Wu Wei Lü Xue San	Wang Cha He Ni Er A	Bangjian	Dispelling wind and eliminating dampness
40	Er Shi Wu Wei Ma Ti San	Da Mou Ni Er A	Dida	Anti-tumour
41	Er Shi Wu Wei Mao Ru San	Sang Dang Ni Er A	Jieji	Dispelling wind and eliminating dampness
42	Er Shi Wu Wei Neng Ming San	Sa Le Xie Ni Er A	Dida	Treat eye disease
43	Er Shi Wu Wei Niu Huang Wan	Ge Wang Ni Er A	Jieji, Dida	Clearing heat
44	Er Shi Wu Wei Qing Gang Zhi Wan	Wai Cha He Ni Er A	Bangjian	Heat-clearing and detoxicating

45	Er Shi Wu Wei Sha Hao Wan	Ca Er Wang Ni Er A	Bangjian	Clear and nourish throat
46	Er Shi Wu Wei Shan Hu Wan	Xu Ma Er Ni Er A	Dida	Opening cardiac
47	Er Shi Wu Wei She Xiang San	La Zi Ni Er A	Dida	Anti-anthrax
48	Er Shi Wu Wei Shen Ban Ji Dou San	Da He Xia Ni Er A	Jieji, Bangjian	Detumescence
49	Er Shi Wu Wei Shi Liu San	Sai Zhi Ni Er A	Dida	Warm tonification
50	Er Shi Wu Wei Song Shi Wan	Yu Niang Ni Er A	Dida	Heat-clearing and detoxicating
51	Er Shi Wu Wei Tan Xiang San	Zan Dan Ni Er A	Dida, Bangjian	Dispelling wind and eliminating dampness
52	Er Shi Wu Wei Te Rou San	Xia Qing Ni Er A	Dida, Ganggaqiong	Clearing heat and relieving pain
53	Er Shi Wu Wei Tian Xian Zi San	Lang Tang Ni Er A	Dida	Insecticidal
54	Er Shi Wu Wei Tong Hui Wan	Sang Ta Le Ni Er A	Dida	Moistening lung
55	Er Shi Wu Wei Wu Jing Jie San	Sao Luo Ni Er A	Bangjian	Clearing heat and benefiting lung
56	Er Shi Wu Wei Xun Dao Niu San	Mang Jian Ni Er A	Dida	Antiplague
57	Er Shi Wu Wei Yan Jing San	Zha He Xing Ni Er A	Dida, Jieji	Dispersing stagnated liver
58	Er Shi Wu Wei Yu Ling San	Tang Se Ni Er A	Dida	Relieve pain
59	Er Shi Wu Wei Yu Zi San	Ji Ri Ni Er A	Dida	Hypotensive
60	Er Shi Wu Wei Zhang Ya Cai San	Dou Da Ni Er A	Dida	Clearing heat and benefiting gallbladder
61	Er Shi Wu Wei Zhen Ca Wan	Zhen Ca Le Ni Er A	Bangjian	Dispelling wind and eliminating dampness
62	Er Shi Wu Wei Zheng Zhang Shi San	Dong Ze Ni Er A	Dida	
63	Er Shi Wu Wei Zhu Sha San	Ca Le Ga Er Ni Er A	Dida	Dispelling wind and eliminating dampness
64	Er Shi Wu Zhu San	Zuo Wo Ni Er A	Dida	Clearing heat
65	Er Shi Yi Wei Han Shui Shi San	Gui Xi Ni Er Jiu	Dida	Clearing lung-heat
66	Fei Yao Bai Se Shang Yin San	Luo Man Ga Er Bao Ya Er Zhen	Bangjian	Reduce phlegm
67	Feng Shi Zhi Tong San	Zhen Bu Sou Jiao He	Dida	Relieve pain
68	Gan Lu Liang Jing San	Dui Zi Si Xing	Dida	Heat-clearing and detoxicating
69	Gan Lu Ling San	Dui Zi Lai Bu Que He	Dida	Clearing heat and relieving pain
70	Han Pei Han Shui Shi San	Gui Xi Zhang Jiao Er	Dida	Heat-clearing and detoxicating
71	He Zi Shuan Hou Ji	A Ri Zhen Xie	Jieji, Bangjian	Clear and nourish throat
72	Huang Yao Pu An San	Man Sai Er Dai Wa Ji Nian	Dida	Tonifying
73	Jiu Pei Feng Mi San	Zhang Jiao Er Ge Wa	Bangjian	Clear and nourish throat
74	Jiu Wei A Zha He San	A Zha He Ju Ge	Dida	Heat-clearing and detoxicating
75	Jiu Wei Ding Xiang San	Le Xi Ge Wa	Bangjian	Heat-clearing and detoxicating
76	Jiu Wei Huang Pei San	Sai Er Bao Ge Jiao Er	Jieji	Anti-infectious disease
77	Jiu Wei Mao Ru Yao Su You	Sang Dang Ge Wei Man Ma Er	Jieji	Dispelling wind and eliminating dampness
78	Jiu Wei Niu Huang San	Ge Wang Ge Wa	Dida	Clearing liver-heat
79	Jiu Wei Tan Xiang San	Zan Dan Ge Wa	Dida	Clearing kidney-heat
80	Jiu Wei Zhang Ya Cai San	Dou Da Ge Wa	Dida	Clearing heat and benefiting gallbladder
81	La Long Hei Yao	La Long Man Na He	Dida	Expelling parasite
82	Lei Feng Tu Ji	Zhen Bu Xiu Man	Dida	Dispelling wind and eliminating dampness
83	Liang Pei Han Shui Shi San	Gui Xi Se Jiao Er	Dida	Detoxification
84	Liang Yue Hui Liu San	Zhan Jiao Mu Da Se	Dida	Clearing heat
85	Liu Wei Bai Hua Qin Jiao San	Ji Jie Zhou Ba	Jieji	Heat-clearing and detoxicating
86	Liu Wei Da Jian Ju Tang San	A Xia He Zhou Tang	Jieji	Clearing lung-heat
87	Liu Wei Ding Xiang San	Le Xi Zhou Ba	Bangjian	Heat-clearing and detoxicating
88	Liu Wei Long Dan Tang San	Bang Jian Zhou Tang	Bangjian	Clear and nourish throat
89	Liu Wei Tu Er Cao Tang San	Hong Lian Zhou Tang	Jieji	Clearing heat
90	Liu Wei Yuan Sui San	E Si Zhou Ba	Jieji	Detumescence
91	Liu Wei Za Mao Lan Zhong Hua	Ao Ne Bu Zhou Tang	Dida	Relieve itching

	Tang San			
92	Liu Wei Zhang Ya Cai San	Dou Da Zhou Ba	Dida	Heat-clearing and detoxicating
93	Liu Wei Zhang Ya Cai Tang San	Dou Da Zhou Tang	Dida	Heat-clearing and detoxicating
94	Long Dan Du Wei Tang	Bang Jian Qiu Tang	Bangjian	Cure black pox
95	Ma Zhen Da Tang San	Si Bu Tang Qing Mao	Dida	Antimeasle
96	Mi Jue Liang Pei San	Man E Se Jiao Er	Dida	Heat-clearing and detoxicating
97	Mi Jue Shi San Wei Hong Hua San	Man Ge Er Ju Song	Dida	Clearing heat and relieving pain
98	Mi Jue Shi Wu Wei Long Dan San	Man E Bang Jian Jiao A	Bangjian	Clearing heat and benefiting lung
99	Mu Bu Zhan Sheng San	Mu Bu Yu Jie	Dida	Clearing stomach-heat
100	Neng An Jun1 Ning San	Dai Xie Ning Dan	Dida	Reduce phlegm
101	Pu Li San	Pan Ba Geng Dan	Dida	Insecticidal
102	Qi Pei Qin Pi San	Da Bu Sang Tun Jiao Er	Dida	Set a broken bone
103	Qi Pei Ya Zui Hua San	Wa Xia Ga Tun Jiao Er	Dida	Clearing heat
104	Qi Pei Zi Cao Rong San	Jia Jie He Tun Jiao Er	Jieji	Detoxification
105	Qi Wei An Lü Zi Jin Tang San	Dai Wa Tun Tang	Jieji	Clearing heat
106	Qi Wei Bing Pian San	Ga Bu Er Tun Ba	Dida	Relieve pain
107	Qi Wei Ci Bai San	Xiu Ci Er Tun Ba	Dida	Tonifying the kidney
108	Qi Wei Da Jian Ju San	Han Er Jiao Mu Tun Ba	Jieji	Relieve pain
109	Qi Wei Da Tang San	Tang Qing Tun Ba	Dida	Clearing heat
110	Qi Wei Ding Xiang San	Le Xi Tun Ba	Dida	Tonifying spleen
111	Qi Wei Du Yi Wei San	Ba He Tun Ba	Jieji, Dida	Clearing heat
112	Qi Wei Duo Sui Liao San	Ni A Luo Tun Ba	Jieji	Clearing heat
113	Qi Wei Gui Pi Tang San	Xiang Ca Tun Tang	Dida	Cure Witbane
114	Qi Wei He Mao Feng Mao Ju Tang San	Za Chi Tun Tang	Jieji	Cholagogic
115	Qi Wei Hong Hua Shu Sheng San	Ge Er Geng Que He Tun	Dida	Heat-clearing and detoxicating
116	Qi Wei Hu Er Cao San	Si Mu Dou Tun Ba	Jieji	Heat-clearing and detoxicating
117	Qi Wei Huang Mao Cui Que Hua San	Gao Le Bei Tun Ba	Jieji	Clearing heat
118	Qi Wei Jiu Hou San	Gao Zhai Ju Zhai Hou Tun	Dida	Warm tonification
119	Qi Wei Kuan Jin Teng Tang San	Lie Zhe Tun Tang	Dida	Heat-clearing and detoxicating
120	Qi Wei Mao Ru Yao Su You	Sang Dang Tun Bei Man Ma Er	Jieji	Clear heat and disinhibit dampness
121	Qi Wei Mao Tang San	Jie Ca Tun Tang	Jieji	Clear heat and disinhibit dampness
122	Qi Wei Mi Guo Qin Tang San	Jia Wa Tun Tang	Jieji	Clear heat and disinhibit dampness
123	Qi Wei Peng Xiao Tang San	Ze Ca Tun Tang	Jieji	Regulating menstruation
124	Qi Wei Qiang Wei San	Gao Le Tun Ba	Jieji	Clear heat and disinhibit dampness
125	Qi Wei Qin Jiao Tang San	Ji Jie Tun Tang	Jieji	Detumescence
126	Qi Wei San Guo Tang San	Zhe Song Tun Tang	Bangjian	Clear and nourish throat
127	Qi Wei Sha Hao San	Ca Er Wang Tun Ba	Bangjian	Clearing heat and benefiting lung
128	Qi Wei Tan Xiang Wan	Zan Dan Tun Ba	Jieji	Clearing heat and benefiting lung
129	Qi Wei Wu Nu Long Dan San	Gang Ga Qiong Tun Ba	Jieji, Ganggaqiong	Anti-diarrhoeal
130	Qi Wei Zhang Mao Feng Mao Ju Tang San	Ao Da Bu Sang Tun Tang	Jieji	Clearing heat
131	Qi Wei Zhang Ya Cai San	Dou Da Tun Ba	Dida	Heat-clearing and detoxicating
132	Qi Zhi San	Yuan La He Tun Dan Ji Jiao Er Wa	Jieji, Bangjian	Insecticidal
133	Ri Yue Ni Hang San	Ni Da Jin Duo	Dida	Secure essence
134	San Bao San	Sou Qing Song Zhao Le	Ganggaqiong	Detoxification
135	San Hua Tang San	Mai Duo Song Tang	Bangjian	Anti-anthrax
136	San Shi Wu Si Wei He Zi San	A Ri Sao A	Dida	Tonifying kidney and diuresis
137	San Shi Yi Wei Chen Xiang San	A Ga Er Sao Jiu	Dida	Dispelling wind and eliminating dampness
138	San Wei He Zi San	A Ri Song Ba	Dida	Relieve pain
139	San Wei Leng Sha Bei Mu Tang San	A Bu Song Tang	Jieji	Dispersing stagnated liver

140	San Wei Long Dan San	Bang Jian Song Tang	Bangjian	Clearing heat
141	San Wei Long Gu San	Zhou Ri Song Ba	Dida	Relieve pain
142	San Wei Qin Jiao Wan	Ji Jie Song Ba	Jieji, Bangjian	Clear and nourish throat
143	San Wei Tou Tang San	Gao Tang Song Ba	Dida	Relieve pain
144	San Wei Zhang Ya Cai Tang San	Dou Hui Song Tang	Jieji, Dida	Clearing heat
145	San Wei Zhu Huang San	Ju Gang Song Ba	Bangjian	Cure esophageal disease
146	Sheng Yao Wang	Ga Le Man Jie Bao	Bangjian	Clear and nourish throat
147	Shi Ba Da Jiao	Jia Er Qing Jiao Jie	Jieji	Dispelling wind and eliminating dampness
148	Shi Ba Wei Bing Pian San	Ga Bu Er Jiao Jie	Dida	Clearing heat
149	Shi Ba Wei Da Xiang San	Lang Qing Jiao Jie	Dida	Warm tonification
150	Shi Ba Wei He Zi Li Niao San	Jin Ni A Ri Jiao Jie	Dida	Tonifying the kidney
151	Shi Ba Wei He Zi San	A Ri Jiao Jie	Dida	Clearing kidney-heat
152	Shi Ba Wei Jin Zhi San	Sai Er Zi Jiao Jie	Dida	Clearing stomach-heat
153	Shi Ba Wei Qiang Wei Hua San	Sai Mai Jiao Jie	Dida	Clearing heat
154	Shi Ba Wei Shi Liu San	Sai Zhi Jiao Jie	Dida	Improve digestion
155	Shi Er Liang San	Sang Bao Ju Ni	Bangjian	Tonifying
156	Shi Er Wei Qi Tang San	Chi Tang Ju Ni	Jieji	Clearing heat
157	Shi Er Wei Tu Er Cao San	Hong Lian Ju Ni	Dida	Antiplague
158	Shi Er Zhu San	Zuo Wo Ju Ni	Dida	Clearing heat
159	Shi Jiu Lei San	Na Mu Zhang Ju Ge	Dida	Tonifying spleen
160	Shi Jiu Wei Cao Guo San	Gao La Ju Ge	Dida	Tonifying spleen
161	Shi Jiu Wei Da Xiang San	Lang Qing Ju Ge	Dida	Detumescence
162	Shi Jiu Wei Shi Liu San	Sai Zhi Ju Ge	Dida	Warm tonification
163	Shi Lei Sheng	Za Mu Lang Zhou Zha	Bangjian	Clear and nourish throat
164	Shi Liu Wei Ye Niu Jiao San	Zhong Ri A Ju Zhou	Dida	Anti-tumour
165	Shi Liu Wei Zhang Ya Cai San	Dou Da Ju Zhou	Dida	Clearing heat
166	Shi Qi Wei Da Xiang Hua San	Lang Qing Mai Duo Ju Tun	Dida	Anti-tumour
167	Shi Qi Wei Shi Hui Yan San	Duo Xing Ju Tun	Jieji	Detumescence
168	Shi San Ceng San	Ri Mu Ba Ju Song	Jieji, Dida	Clearing heat
169	Shi San Wei Bai Se Shang Yin Wan	Ga Er Bao Ya Er Zhen Ju Song	Bangjian	Clearing heat and benefiting lung
170	Shi San Wei Cao Tang San	Ao Tang Ju Song	Dida	Detoxification
171	Shi San Wei Da Xiang San	Lang Qing Ju Song	Dida	Hemostatic
172	Shi San Wei Hei Bai Wan	Ga Er Na He Ju Song	Dida	Clearing heat
173	Shi San Wei Hua Er San	Mai Duo Ju Song	Dida	Heat-clearing and detoxicating
174	Shi San Wei Lang She San	Jiang Jie Ju Song	Bangjian	Heat-clearing and detoxicating
175	Shi San Wei Long Dan San	Bang Jian Ju Song	Bangjian	Clearing heat and benefiting lung
176	Shi San Wei Qian Li Guang San	You Xiang Ju Song	Dida	Detoxification
177	Shi San Wei Shang Yin San	Ya Er Zhen Ju Song	Bangjian	Reduce phlegm
178	Shi San Wei Tang Gu Te Wu Tou San	Wang Ga Er Ju Song	Jieji, Dida	Heat-clearing and detoxicating
179	Shi San Wei Xie Dan San	Chi Gui Ju Song	Dida	Purgation
180	Shi San Wei Ying Xiong San	Hua Wo Ju Song	Jieji	Anti-measles
181	Shi San Wei Zhang Ya Cai San	Dou Da Ju Song	Dida	Clearing heat and benefiting gallbladder
182	Shi San Wei Zhu Huang Wan	Ju Gang Ju Song	Bangjian	Reduce phlegm
183	Shi Si Ceng San	Ri Mu Ba Ju Yu	Jieji	Clearing heat
184	Shi Si Wei Bing Pian San	Ga Bu Er Ju Yu	Dida	Anti-influenza
185	Shi Si Wei Peng Niao San	Xia Qiong Ju Yu	Dida	Insecticidal
186	Shi Si Wei Tang Gu Te Wu Tou San	Wang Ga Er Ju Yu	Jieji, Bangjian	Clearing heat
187	Shi Wei Bai Hua Qin Jiao San	Ji Jie Ju Wa	Jieji	Heat-clearing and detoxicating
188	Shi Wei Da Tang San	Tang Qing Ju Wa	Dida	Clearing heat and relieving pain
189	Shi Wei He Zi San	A Ri Ju Wa	Dida	Promoting blood circulation and removing blood stasis

190	Shi Wei He Zi Tang San	A Ri Ju Wa	Dida	Clearing heat
191	Shi Wei Ling Gai San	Tuo Le Ri Ju Wa	Dida	Clearing heat
192	Shi Wei Long Dan San	Bang Jian Ju Wa	Bangjian	Clearing heat and benefiting lung
193	Shi Wei Nao Sha San	Jia Ca Ju Wa	Bangjian, Ganggaqiong	Detoxification
194	Shi Wei Niu Huang San	Ge Wang Ju Wa	Dida	Clearing heat
195	Shi Wei Zhen Ca San	Zhen Ca Le Ju Wa	Dida	Relieve pain
196	Shi Wu Wei Bang Cha He San	Bang Cha He Jiao A	Bangjian	Clearing heat and benefiting lung
197	Shi Wu Wei Han Shui Shi San	Gui Xi Jiao A	Dida	Anti-tumour
198	Shi Wu Wei Hei Bai Wan	Ga Er Na He Jiao A	Dida	Relieve pain
199	Shi Wu Wei Liang Jing San	Se Ji Le Jiao A	Dida	Sedative
200	Shi Wu Wei Long Dan San	Bang Jian Jiao A	Bangjian	Clearing heat and benefiting lung
201	Shi Wu Wei Shui Bai Zhi Tang San	Ao Mu Tang Jiao A	Ganggaqiong	Heat-clearing and detoxicating
202	Shi Wu Wei Zhang Ya Cai San	Dou Da Jiao A	Dida, Jieji	Clearing heat
203	Shi Yi Wei Da Xiang San	Lang Qing Ju Jiu	Dida	Clearing heat
204	Shi Yi Wei Ding Xiang San	Le Xi Ju Jiu	Bangjian	Moistening lung
205	Shi Yi Wei Lei Feng Tang San	Zha He Tang Ju Jiu	Jieji	Dispelling wind and eliminating dampness
206	Shi Yi Wei Nao Sha San	Jia Ca Ju Jiu	Ganggaqiong, B angjian	Detoxification
207	Shi Yi Wei Niu Huang Wan	Ge Wang Ju Jiu	Dida	Clearing heat
208	Shi Yi Wei Qing Gang San	Wai Duo Ju Jiu	Jieji	Clearing heat
209	Shi Yi Wei Tan Xiang San	Zan Dan Ju Jiu	Dida	Tonifying the kidney
210	Shi Yi Wei Tang Gu Te Wu Tou San	Wang Ga Er Ju Jiu	Dida	Detoxification
211	Shi Yi Wei Wu Jing Jie San	Sao Luo Ju Jiu	Bangjian	Clearing heat and benefiting lung
212	Shi Yi Wei Zhi Xie Mu Zi San	Dou Niang Ju Jiu	Dida	Clearing heat and benefiting gallbladder
213	Shi Yi Wei Zi Cao Rong San	Jia Jie He Tun Ju Jiu	Bangjian	Clearing heat and benefiting lung
214	Si Wei Hong Hua Tang San	Ge Er Geng Yu Tang	Dida	Detoxification
215	Si Wei Long Dan Tang San	Bang Jian Yu Tang	Bangjian	Clear and nourish throat
216	Si Wei Mao Ru Tang San	Sang Dang Yu Tang	Jieji	Clear heat and disinhibit dampness
217	Si Wei Pu Tao Tang San	Geng Zhen Yu Tang	Jieji	Relieve pain
218	Si Wei Tang Gu Te Wu Tou San	Wang Ga Er Yu Tang	Bangjian	Clearing heat
219	Si Wei Tu Er Cao San	Hong Lian Yu Wa	Dida	Clearing heat
220	Si Wei Zhang Ya Cai Tang San	Dou Hui Yu Tang	Dida	Clearing heat
221	Wang Pei Zhi Gen San	Dang Tun Jia Wei Jiao Er Wa	Dida	Clearing heat
222	Wu Si Liang Yue San	Da Se Qu Mai	Dida	Relieve pain
223	Wu Si Sheng	Qu Mai Xia He Bai Jiao Er Wa	Dida	Anti-influenza
224	Wu Wei Bai Hua Qin Jiao San	Ji Jie A Wa	Jieji	Heat-clearing and detoxicating
225	Wu Wei Duan Sui Tu Er Cao Tang San	Sa Zeng A Tang	Jieji	Hemostatic
226	Wu Wei Kuan Jin Teng Tang San	Lie Zhe A Tang	Dida	Dispelling wind and eliminating dampness
227	Wu Wei Run Jiang Tang San	Ling Lun A Tang	Dida	Clearing heat
228	Wu Wei Tang Gu Te Wu Tou Tang San	Wang Ga Er A Tang	Bangjian	Clear and nourish throat
229	Wu Wei Wu Tou Tang San	Wang A A Tang	Bangjian	Clear and nourish throat
230	Wu Wei Zhang Ya Cai Tang San	Dou Da A Tang	Dida	Clearing liver-heat
231	Wu Yun Yue Guang San	Zhen Zha Le Da Sai Er	Dida	Sedative
232	Xue Ya Jun Ning San	Cha He Xie Nian Dan	Dida	Hypotensive
233	Yi Chu Neng An Da Jiao	Jia Er Qing Re Ba Dai Xie	Jieji	Dispelling wind and eliminating dampness
234	Ying Xiong Pu Mie San	Zha He Bao Geng De Le	Dida	Clearing heat
235	Yu Tuo Si Pei Cao Yao	Yu Tuo He De Mu Yu Jiao Er Wa	Dida	Clearing heat
236	Yue Jing Gan Lu Di	Da Xie Shi Dui Zi Te Ba	Dida	Heat-clearing and detoxicating
237	Yue Jing Miao San	Da Xie Ni Ge	Dida	Heat-clearing and detoxicating

238	Zhang Ya Cai Du Wei Tang	Dou Da Qiu Tang	Dida	Clearing heat
239	Zhang Ya Cai Jiu	Dou Da Qiang	Dida	Clearing heat
240	Zhen Bao Chu Han Pei	Ren Qing Zhang Jiao Er	Jieji, Dida, Bangjian, Ganggaqiong	Detoxification
241	Zhen Bao Er Shi Wu Wei Shan Hu	Ren Qing Xu Ma Er Ni Er A	Dida	Opening cardiac
242	Zhen Bao Er Shi Wu Wei Song Shi Wan	Ren Qing Yu Niang Ni Er A	Dida	Heat-clearing and detoxicating
243	Zhen Bao Sheng Pei	Ren Qing Xia He Bei Jiao Er Wa	Dida	Anti-influenza

Table 3 Iridoids from Gentianeae

Table 4 Xanthones from Gentianeae

Table 5 Flavonoids from Gentianeae

Table 6 Triterpenoids from Gentianeae
